# Shikonin Inhibits Fin Regeneration in Zebrafish Larvae

**DOI:** 10.3390/cells11203187

**Published:** 2022-10-11

**Authors:** Zigang Cao, Chen Guo, Guilan Chen, Jiejun Liu, Huiwen Ni, Fangsheng Liu, Guanghua Xiong, Xinjun Liao, Huiqiang Lu

**Affiliations:** Jiangxi Engineering Laboratory of Zebrafish Modeling and Drug Screening for Human Diseases, Jiangxi Key Laboratory of Developmental Biology of Organs, College of Life Sciences, Jinggangshan University, Ji’an 343009, China

**Keywords:** shikonin, fin regeneration, AMPK, zebrafish

## Abstract

Shikonin is a naphthoquinone compound extracted from Chinese comfrey for treating cancer. However, there are few reports on its research on vertebrate tissue regeneration. Zebrafish is an ideal model for studying organ regeneration. In this study, we found that 3-dpf of zebrafish larvae exposed to shikonin at concentrations of 0.2, 0.3, and 0.4 mg/L showed increasingly inhibited regeneration of the tail fin. Immunohistochemical staining showed that shikonin exposure from 6 to 12 hpa increased the number of apoptotic cells in the caudal fin wound of larvae and decreased the number of proliferating cells. Shikonin exposure was found to up-regulate oxidative stress, increase ROS levels, and reduce neutrophil recruitment in the early stage of wound repair. Moreover, shikonin exposure caused disordered expression of fin regeneration blastemal-related genes. The use of astaxanthin to down-regulate oxidative stress was found to significantly reduce the inhibition of caudal fin regeneration. Mixed exposure of AMPK inhibitors or fullerenes (C60) with shikonin also showed the similar rescue effect. Collectively, our study showed that shikonin inhibited fin regeneration in zebrafish larvae by the upregulation of oxidative stress level and AMPK signaling pathway. This research provides valuable information on the mechanism of action of shikonin for its safe application.

## 1. Introduction

Shikonin comfrey is a perennial herbaceous plant in the Boraginaceae family. Oils and ointments with shikonin as the main ingredient have been used clinically in China, Japan, and Europe for the treatment of burns and as skin care products [1]. In addition, shikonin is used as a food additive in many countries. Lithospermum dry extract has been used in traditional Chinese medicine to treat various diseases including inflammation and cancer, and the key active substance in the extract is naphthoquinone shikonin [1]. In recent years, shikonin has been proven to be useful for treating obesity, inhibiting the proliferation of human lung adenocarcinoma cells, inducing apoptosis and autophagy in human liver cancer and pancreatic cancer cells, and reducing immune rejection of allogeneic organs [2,3,4,5,6]. However, the effect and mechanism of shikonin on tissue regeneration have not yet been reported yet.

Zebrafish (*Danio rerio*) genome is 87% homologous to humans and have powerful regenerative capacity [7]. Zebrafish fin regeneration is a good model for studying limb regeneration and the mechanism of limb regeneration is highly conserved in the evolution of vertebrates [8]. Many types of cells are involved in the fin regeneration process, including immune cells, nerve cells, mesenchyme cells, skin cells, and bone cells [9]. Neutrophils play a key role in the response to fin injury. As the “first responder” to tissue injury, they accumulate at the wound site at the early stages of inflammatory response and remain the most abundant cells in the first 24 h [10]. Macrophages actively participate in all stages of acute wound healing and are essential for fin regeneration [11]. Fin regeneration is also regulated by many factors and signaling pathways, such as Fgf, Wnt, and RA [12,13]. Thus, zebrafish fin regeneration model has widely been accepted as an ideal model for studying the effects of chemicals on wound healing and tissue regeneration.

In this study, we found that shikonin can inhibit the regeneration of caudal fin in zebrafish larvae. Experiments showed that shikonin exposure can significantly increase the number of apoptotic cells in the wound and upregulate oxidative stress level and apoptosis-related genes. The oxidative stress inhibitors, fullerene (C60) and astaxanthin, can rescue the phenotype of inhibited tail fin regeneration. This study clarified the possible toxicological effects and mechanisms of shikonin and provided effective intervention methods.

## 2. Materials and Methods

### 2.1. Fish Strain

Zebrafish wild-type (AB), *Tg (lyz:DsRed)* and *Tg (coro1a:GFP)* transgenic strains obtained from China Zebrafish Resource Center (Wuhan, China) were used. All fish were maintained in a circulation culture system of 28 ± 1 °C, with a photoperiod of 14 h: 10 h light/dark, and fed with Artemia. All fish were reared and maintained under standard laboratory conditions in accordance with experimental guidelines. Zebrafish embryos were cultured with methyl blue fish fluid between 0 and 24 h post fertilization (hpf). At 24 hpf, embryo culture containing 0.003% PTU (1-phenyl−2-thiourea, Sigma-Aldrich, Darmstadt, Germany) was used to inhibit skin pigment production.

### 2.2. Chemical

Shikonin analysis reference substance was purchased from Beijing Soleibao Technology Co., Ltd., Beijing, China. RNA extraction kit Tranzol UP (ET111–01), cDNA Transcription Kit (AE311–03) and TransStart^@^ Tip Green qPCR Supermix (AQ601–02) were purchased from Beijing Quanjin Biotechnology Co., Ltd. Reactive oxygen detection kits were purchased from Nanjing Jiancheng Institute of Bioengineering (Nanjing, China). TUNEL Apoptosis Detection Kit (Alexa Fluor 640) was purchased from Yeasen Company (Shanghai, China).

### 2.3. Fin Amputation and Drug Treatments

**Figure 1:**
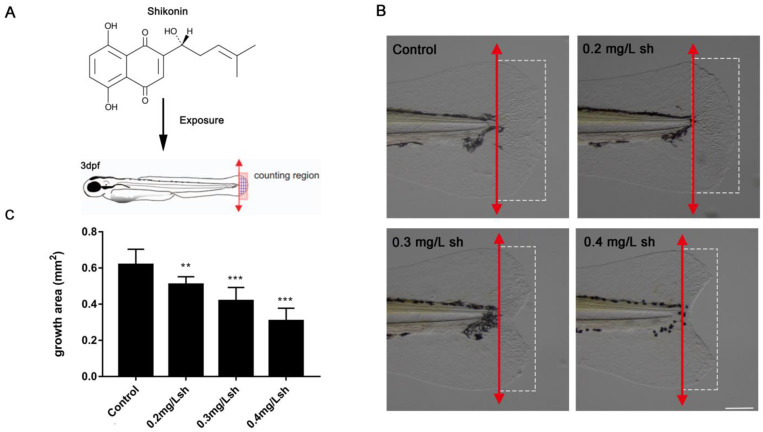
Shikonin (sh) exposure inhibits fin regeneration in zebrafish larvae. (**A**). Schematic diagram of shikonin treatment in zebrafish fin regeneration. The red double arrow marks the specific excision site of the caudal fin, and the blue reticulated area represents the statistical region of the regeneration area. (**B**). Zebrafish larvae exposed to 0.2 (n = 10/15), 0.3 (n = 13/15), and 0.4 (n = 15/15) mg/L shikonin for 48 hpa after tail-cutting at 3 dpf. The red double arrow shows the excision site of the caudal fin. (**C**). Quantification of 48 hpa fin regeneration area (mm^2^) (n = 8). Data represents the mean ± S.D. standard deviation ** *p* < 0.01, *** *p* < 0.001. Scale bar: 100 μm.

Fin amputation and drug treatments were performed as described in References [14,15]. The caudal fins of 3 dpf larvae were amputated (just posterior to the notochord) with sterilized blades (Figure 1A), and fish were then transferred in a six-well polystyrene plate, with 15 fish in each well, and allowed to regenerate in shikonin solutions or shikonin-free embryonic medium (EM) for various times. In each experiment, four different treatment groups were used: a vehicle (DMSO), three treatment groups (0.2, 0.3, and 0.4 mg/L shikonin) All groups were incubated at 28.5 °C and the treatment reagents were replaced every 24 h. The morphology of the tail fin was observed under a microscope at 6 hpa, 12 hpa, 48 hpa, and 72 hpa. Then, larvae were collected for subsequent experimental analysis. All experiments were repeated three times for each concentration. The larvae processed in each group at different time periods were anesthetized with 0.16% tricaine and fixed using 1% low-melting agarose. A SteREO DiscoveryV20 microscope (Carl Zeiss, Germany) equipped with AxioVision Rel 4.8.2 software was used to take a phenotype map and measure the length and area of the regenerated fin.

### 2.4. Gene Transcription Level Analysis

At 48 hpa, 60 larvae were taken from each group; their tails were cut off and collected, then rinsed 3 times with PBS buffer before the extraction of total RNA with Trizol reagent (Invitrogen). Only samples with an RNA 260 nm/280 nm ratio between 1.8 and 2.0 were used. cDNA was synthesised with cDNA reverse transcription reagents, TransStart^@^ Tip Green qPCR Supermix (AQ601-02). Real-time quantitative PCR detection of genes was performed using an ABI Step One plus RT-PCR system. Genes related to primordium development (lef1, fgf20a, osn, mxsb), cell survival factor (bcl2), apoptosis factor (bax), tumour suppressor (p53), downstream genes of oxidative stress (glipr1a-1, nox1-1, glipr1b-2) were measured. The primers were sourced from [4,5,6,9]. For each gene, β-actin was used as an internal reference, and the relative RNA expression level was calculated using the 2^−ΔΔCt^ method.

### 2.5. Antibody, TUNEL, ROS and Cridine Orange (AO) Staining

Samples were taken using 1.5 mL EP tubes, fixed with 4% PFA (paraformaldehyde) overnight, and then made transparent using acetone at −20 °C overnight (or at 4 °C for 6 h). The samples were then rinsed with 3% PT (3% TritonX-100, PBS, PH ≈ 7) for 2 to 3 times, blocked with PBTN (4% BSA, 0.02% NaN_3_, 3% PT) 2 h, followed by the addition of PCNA antibody (1:500, ab71286, Abcam, Cambridge, UK) and left overnight at 4 °C. The primary antibody was recovered on the third day, and the samples rinsed 5 times again with PT for 45 min each time. A secondary antibody (1:2000, Carlsbad, CA, USA) was then added and left overnight at 4 °C. On the fourth day, the secondary antibody was recovered and the sample was rinsed again with PT, then counterstained with DAPI, Samples were placed in DAPI dye solution (5 µg/mL), incubated at 37 °C for 30 min, and rinsed 3 times with PT, and finally was imaged with a laser scanning confocal microscope.

TUNEL staining was performed following the manufacturer’s instructions. The sample was digested with 20 μm/mL protease K for 35 min, rinsed with PBS for 3 times. The positive control group was incubated with 1× DNase I Buffer for 25 min, then the Buffer was removed and 10 U/mL DNase I was added for digestion for 10 min (only the positive control group needed DNase I incubation. The experimental group did not need it). Then, the samples were incubated with 1× Equilibration Buffer for 30 min, followed by further incubation in TdT buffer for 60 min, and then washed with PBTN for 2 to 3 times, and finally stained with DAPI, and imaged with a laser scanning confocal microscope (Leica TCS SP8, Wetzlar, Germany).

Acridine orange (AO) staining was used to detect apoptotic cells. Fifteen samples were collected and exposed to the configured ROS dye (H2DCFDA:0.003% PTU solution = 1:1000), and incubated at 37 °C in darkness for 30 min, and then rinsed with 0.003% PTU for 5 times in darkness until the fluorescence intensity was unchanged. ROS staining were imaged by using a Leica microscope (M205FA) with the same parameters. The samples were collected, added 5 mg/L AO, incubated at 28 °C for 30 min in darkness, and washed with 0.003% PTU for 3 times. After anesthesia with tricaine, apoptotic cells were photographed with Zeiss stereoscopic microscope. The cell number and fluorescence intensity were quantified by Image J 2022 software.

### 2.6. Rescue Experiment

Wild-type (AB) zebrafish larvae were trimmed at 3 dpf and exposed to a mixture of 0.4 mg/L shikonin and 1.13 μM astaxanthin (Solarbio, Beijing, China, UV ≧ 98%), 600 nM C60 [16], 12 μM vidarabine (MCE, New Jersey, NJ, USA). Control groups were also set up and exposed to a similar mixture for 48 h. Images of the samples were acquired using a Leica M205FA microscope.

### 2.7. Statistical Analysis

All statistical analyses were performed using GraphPad Prism 7.0 version. The Student’s *t*-test was used to determine statistical significance. All data represents the mean ± S.D. standard deviation * *p* ≤ 0.05, ** *p* ≤ 0.01, *** *p* ≤ 0.001.

## 3. Result

### 3.1. Morphological Observation

Shikonin is a naphthoquinone drug, and the molecular formula is illustrated in Figure 1A. In our study, we selected 3 dpf healthy zebrafish larvae with consistent size for tail amputation (Figure 1A) and performed shikonin treatment immediately on operated zebrafish larvae as described in the References [14,15]. We determined the concentrations of shikonin based on the phenotype of defective fin regeneration in zebrafish larvae and the literature described by He et al. [17]. 0.2, 0.3 and 0.4 mg/L shikonin induced mild, moderate, and severe inhibited fin regeneration, respectively (Figure 1B). Additionally, the area of tail fin regeneration gradually decreased with increasing concentration (Figure 1C). The results showed that shikonin inhibits zebrafish larvae fin regeneration in a dose-dependent manner.

### 3.2. Gene Expression Analysis

**Figure 2:**
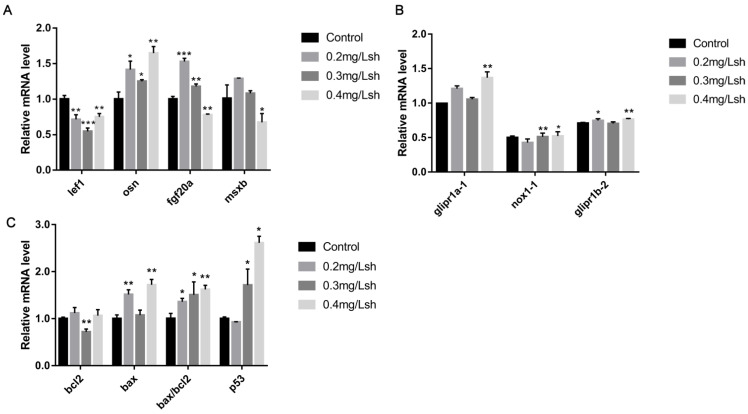
Gene expression analysis. (**A**) Expression analysis of genes related to blastemal formation (**B**) Analysis of gene expression of downstream markers of oxidative stress. (**C**) Real-time qPCR of apoptosis-related genes bax, bcl2, and p53. (All the above experiments used β-actin as the load control. Data expressed as the mean ± SEM of three independent experiments. Data represents the mean ± SD standard deviation * *p* < 0.05, ** *p* < 0.01, *** *p* < 0.001).

We used RT-qPCR to detect the expression levels of genes related to blastemal formation, apoptosis-related genes, and downstream genes of oxidative stress. Compared with the control group, the expression level of lef1 in the shikonin treatment group was significantly reduced, while the expression level of fgf20a increased. The expression of osn and msbx increased at low concentrations and decreased at high concentrations (Figure 2A). The results suggest that shikonin treatment can cause the disorder of the expression of blastemal related genes in zebrafish fin regeneration. The expression levels of apoptosis factors p53, bax and cell survival factor bcl2 were detected. The RT-qPCR results showed that the bax/bcl2 value and p53 level increased significantly (Figure 2C), indicating that shikonin induced apoptosis. At the same time, we found that the expression levels of glipr1a-1 and glipr1b-2, the downstream genes of oxidative stress, increased significantly, and the expression level of nox1-1 decreased (Figure 2B), indicating that shikonin can induce oxidative stress.

### 3.3. Shikonin Induces the Accumulation of ROS in the Tail, Increasing Apoptotic Cells and Decreasing Proliferation

**Figure 3:**
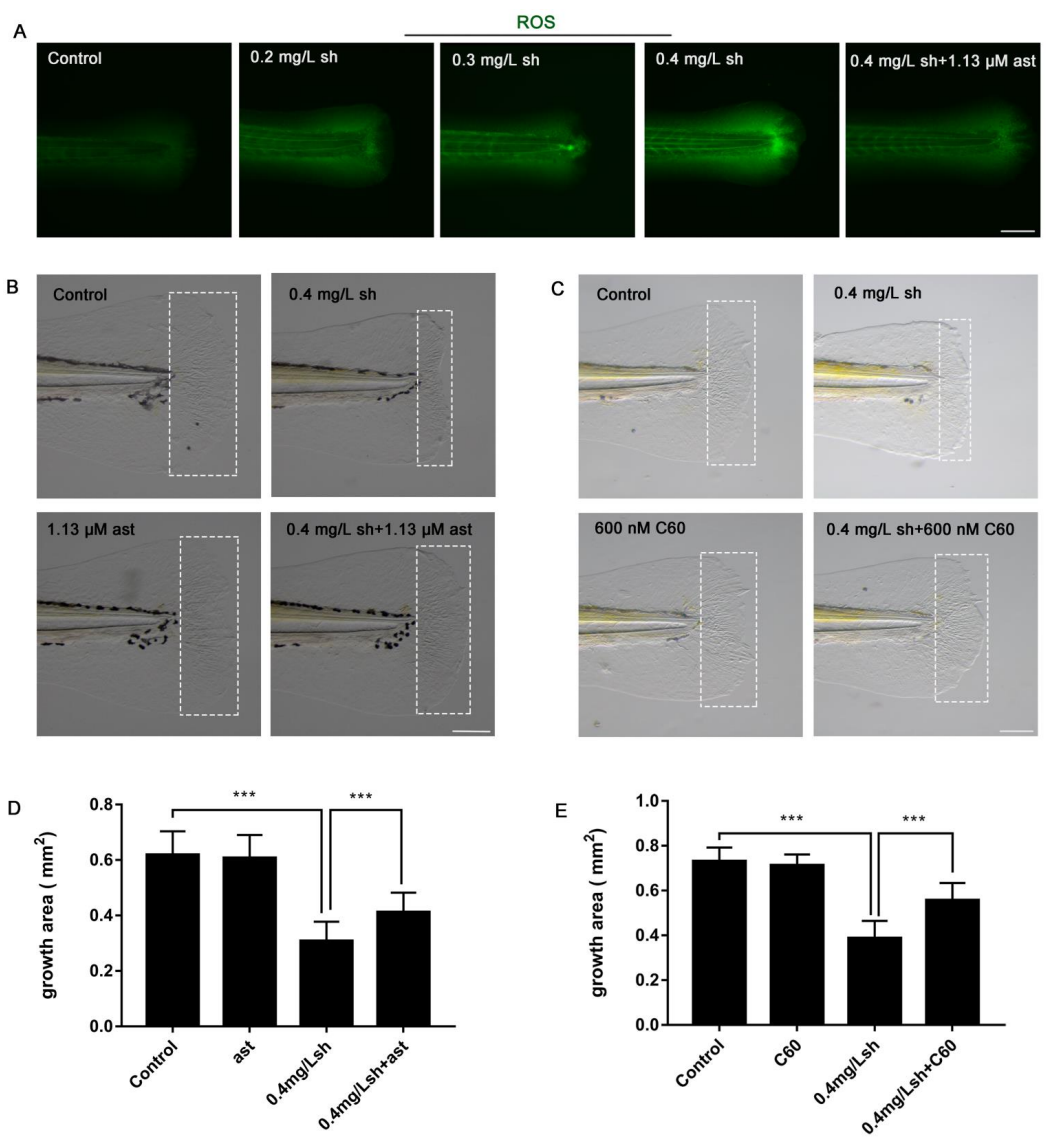
Fullerene and astaxanthin can rescue fin regeneration defects caused by shikonin. (**A**) ROS staining results show that ast (1.13 μM) regulated oxidative stress. (**B**) Astaxanthin rescued defects of fin regeneration caused by shikonin (Control (15/15), 0.4 mg/Lsh (n = 14/15), 1.13 μM ast (14/15), 0.4 mg/Lsh + 1.13μM ast (13/15)). (**C**) Fullerene (C60, 600 nM) rescued shikonin-induced fin regeneration defects. (Control (15/15), 0.4 mg/Lsh (n = 14/15), C60 (600 nM) (14/15), 0.4 mg/Lsh + C60 (600 nM) (13/15)). (**D**,**E**) Quantification of caudal fin regeneration area after astaxanthin (**D**) and fullerene (**E**) rescue at 48 hpa (mm^2^) (n = 9). Data represents the mean ± S.D. standard deviation *** *p* < 0.001. Scale bars: 150 μm (**A**), 100 μm (**B**,**C**).

**Figure 4:**
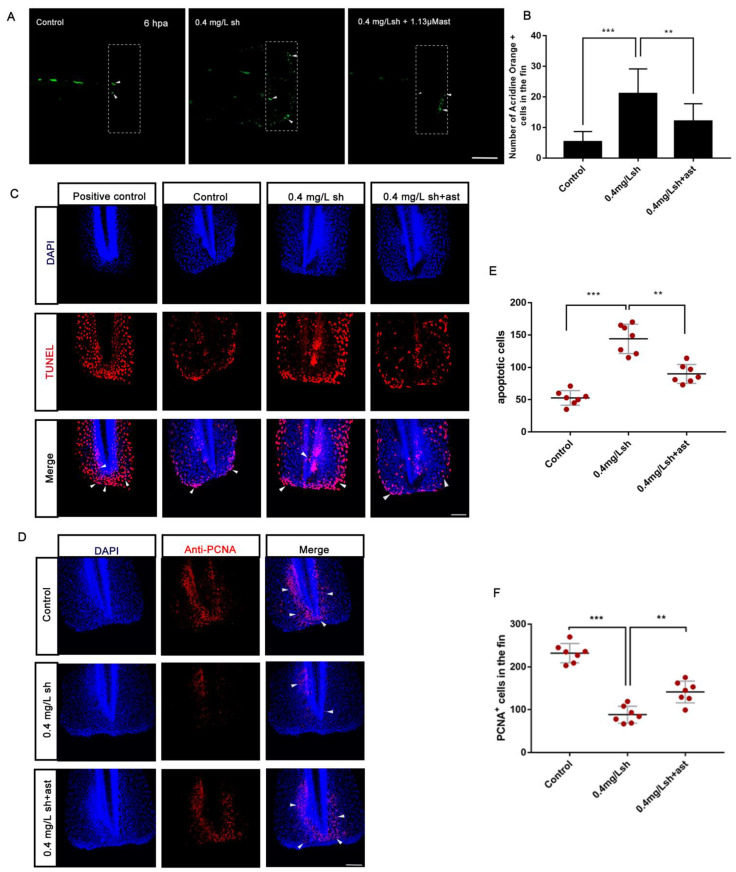
Shikonin promotes the increase of apoptotic cells and the decrease of proliferative cells. (**A**) Acridine orange (OA) staining at 6 hpa. Control (15/15), 0.4 mg/Lsh (n = 14/15), 0.4 mg/Lsh + 1.13μM ast (13/15). (**B**) Statistics of the number of positive cells stained with acridine orange. (**C**) TUNEL staining of fin regeneration at 12 hpa. White arrows mark the apoptotic cells (N = 7). (**D**) PCNA antibody staining of fin regeneration at 24 hpa, white arrows mark the PCNA+ cells (n = 7). (**E**) Quantitative statistics of the number of apoptotic cells (n = 7). (**F**) Quantitative statistics of the number of proliferating cells labelled with PCNA+ (n = 7). Data represents the mean ± S.D. standard deviation ** *p* < 0.01, *** *p* < 0.001. Scale bars: 100 μm (**A**), 75 μm (**C**,**D**).

In order to study the mechanisms of shikonin-induced defects in the regeneration of caudal fin in zebrafish larvae, we evaluated the distribution of reactive oxygen species (ROS) and oxidative stress indicators. Data showed that shikonin induced the production of ROS (Figure 3A). To further confirmed whether shikonin inhibits fin regeneration by inducing oxidative stress, we used oxidative stress inhibitors, astaxanthin and fullerene. Our results showed that astaxanthin and fullerene had little effects on fin regeneration and effectively rescued phenotype of fin regeneration damaged by shikonin (Figure 3A–E). AO staining and TUNEL staining showed a significant increase in the number of apoptotic cells in the tail after shikonin treatment (Figure 4A–C), while the apoptotic cells in the astaxanthin treated group were significantly reduced (Figure 4B,E). The results of PCNA antibody staining at 24 dpa showed that the proliferating cells were significantly reduced after shikonin treatment, and were partially rescued by astaxanthin (Figure 4D,F). These results showed that shikonin increased the number of apoptotic cells and decreased the number of proliferating cells at the early stages of fin regeneration by inducing oxidative stress.

### 3.4. Shikonin Will Reduce the Recruitment of Neutrophils to the Fin Wound

**Figure 5:**
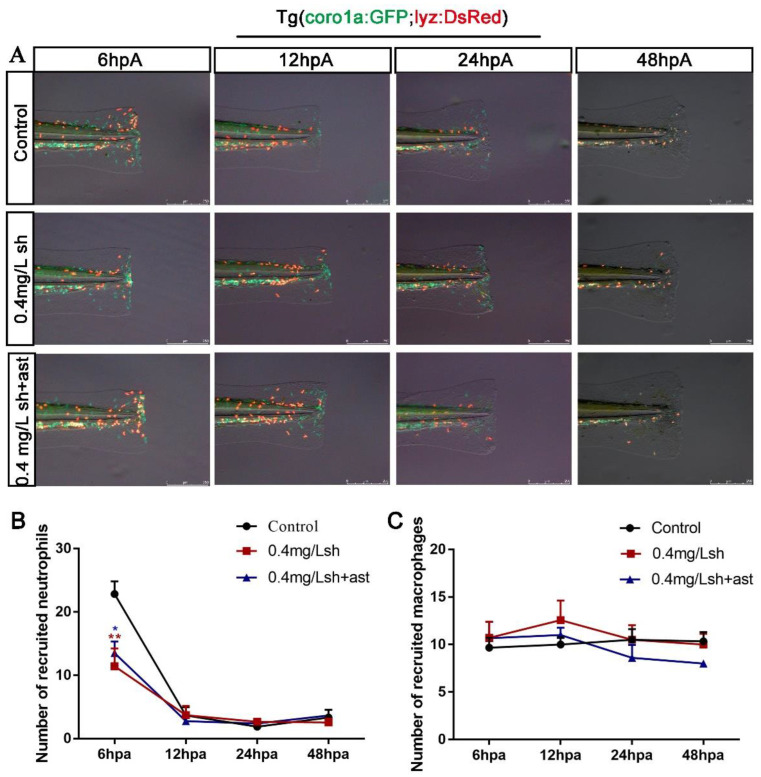
Shikonin reduces the number of neutrophils recruited at the incision. (**A**) Three experimental groups, control, 0.4 mg/Lsh and 0.4 mg/Lsh + 1.13 μM ast (n = 15), were observed under transmitted and fluorescence illumination at 6 hpa, 12 hpa, 24 hpa and 48 hp. (**B**) Statistical analysis of the number of neutrophils at each stage. 0.4 mg/Lsh at 6hpa decreased neutrophils compared to the Control group (** *p* red), and 0.4 mg/Lsh + 1.13 μM ast increased neutrophils compared to the 0.4 mg/Lsh group (* *p* blue). (**C**) Statistical analysis of the number of macrophages at each stage. Data represents the mean ± S.D. standard deviation * *p* < 0.05, ** *p* < 0.01.

In order to explore the effect of shikonin on the recruitment of immune cells in the wound, we used *Tg (lyz:DsRed)* and *Tg (coro1a:GFP)* double transgenic lines for labelling macrophages and neutrophils. The results showed that shikonin treatment at 6 hpa reduced the number of neutrophils in wound, while astaxanthin slightly rescued the number of neutrophils at 12, 24 and 48 hpa (Figure 5A,B). It is known that macrophages actively participate in all stages of acute wound healing and are essential for proper wound healing [11]. In this study, the reduction of wound neutrophils did not affect the activation of macrophages. There was no significant difference in the number of macrophages in each period (Figure 5A,C), and they mainly gathered in the thickened tissue of the wound, which might be caused by the increase of apoptotic cells (Figure 4A,C,E). Our results indicated that shikonin treatment will at least lead to a decrease in the number of early recruitment of neutrophils.

### 3.5. Inhibition of AMPK Signaling Can Partially Rescue the Hindered Tail Fin Regeneration Caused by Shikonin

**Figure 6:**
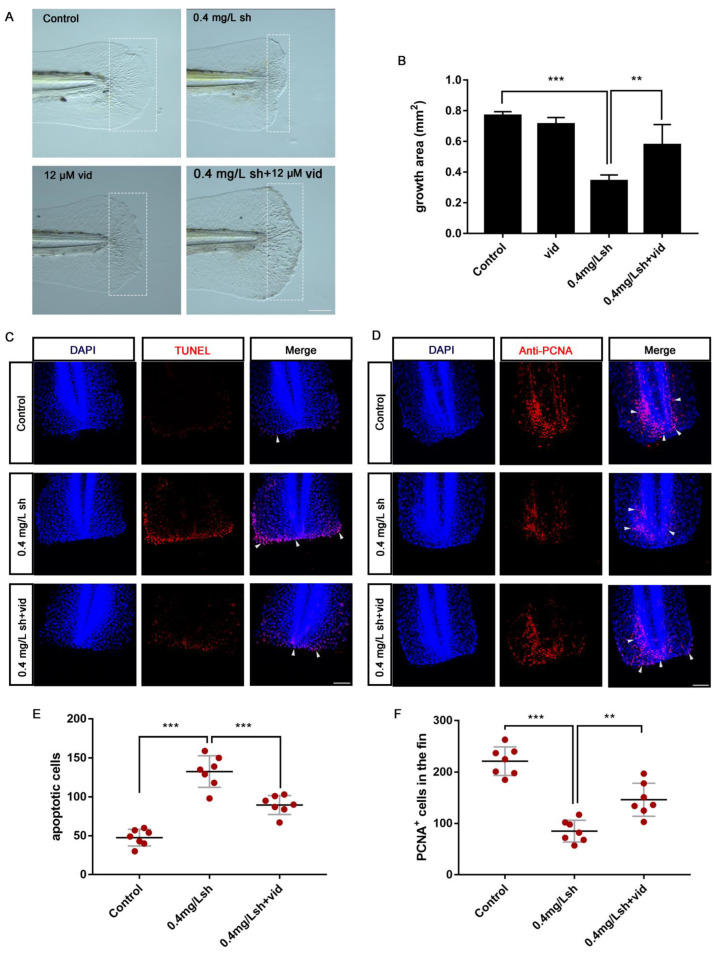
AMPK inhibitor vidarabine (vid) can alleviate hindered tail fin regeneration caused by shikonin. (**A**) White light phenotype after 48 hpa exposure of control, 0.4 mg/Lsh and 0.4 mg/Lsh + 12 μM vid groups. (**B**) Quantification of the regenerated fin area (mm^2^) corresponding to (**A**) (n = 9). (**C**) Confocal images showing TUNEL staining of the tails of fish at 12 hpa, with white arrows marking the apoptotic cells (n = 7). (**D**) Confocal images showing the PCNA antibody staining of the tails of fish at 24 hpa. White arrows show the PCNA+ cells (n = 7). (**E**) Quantitative analysis of the number of apoptotic cells (n = 7). (**F**) Quantitative analysis of the number of proliferating cells labelled with PCNA+ (n = 7). Data represents the mean ± S.D. standard deviation ** *p* < 0.01, *** *p* < 0.001. Scale bars: 100 μm (**A**), 75 μm (**C**,**D**).

AMPK signaling plays an important role in regulating cell metabolism and maintaining cell energy homeostasis and is involved in many other cellular processes such as apoptosis [18,19]. In addition, there is relevant evidence that AMPK plays a crucial role in the survival and growth of tumor cells and is considered as a potential therapeutic target for the treatment of cancer [20,21]. In order to explore whether shikonin inhibits fin regeneration by activation of the AMPK signalling pathway, we exposed the tail-cut zebrafish larvae to AMPK inhibitor vidarabine (vid) together with shikonin. The results showed that vid can partially rescue the hindered tail fin regeneration caused by shikonin (Figure 6A,B). In addition, the results of TUNEL staining and PCNA antibody staining showed that the number of apoptotic cells in the vid treatment group was reduced compared with the shikonin treatment group (Figure 6C,E), while the number of proliferating cells in the vid treatment group was significantly increased compared with the shikonin treatment group (Figure 6D,F). Overall, the results showed that shikonin can activate AMPK signalling pathway and affect zebrafish larvae fin regeneration.

## 4. Discussion

The regeneration process of caudal fin in zebrafish larvae is very similar to that in adult fish [22]. More importantly, the regeneration of caudal fin in zebrafish larvae morphologically completes in three days after amputation. This enables a larger experimental sample size and a shorter experimental time period. Therefore, many researchers use zebrafish larvae caudal fin excision models to evaluate the effects of drugs on wound repair and tissue regeneration. For example, ginsenoside Rg1, which is a natural plant extract like shikonin, has a similar glucocorticoid structure and a good anti-inflammatory effect. Researchers have used the zebrafish larvae caudal fin resection model to evaluate the ginsenoside’s effect on inflammation and regeneration of damaged tissues [17].

In this study, we used 3 dpf zebrafish larvae to study the regeneration effect of shikonin on resected caudal fin between 0 dpa and 72 dpa. Our research results showed that shikonin inhibited fin regeneration in zebrafish larvae (Figure 1B). The mechanism through which shikonin affects wound repair and tissue regeneration was investigated.

Blastemal formation is crucial to the fin regeneration; the blastemal markers such as lef1, fgf20a, osn, and msxb positively regulate blastemal formation and the regeneration process [9]. We evaluated the expression of these genes and results showed that at a shikonin concentration of 0.4 mg/L, only osn expression increased while the other three genes decreased significantly compared to the control group (Figure 2A). The expression level of lef1 gene significantly reduced at all three concentrations of shikonin, while the expression of fgf20a, osn, and msxb increased at low and medium concentrations, and decreased at high concentrations. This may be due to apoptosis caused by oxidative stress in the process of fin regeneration.

Excessive accumulation of ROS can cause damage to cellular components such as lipids, proteins, and DNA, and ultimately lead to pathological conditions [23]. Studies have shown that shikonin induces the production of reactive oxygen species (ROS) in cells, depolarizes the mitochondrial membrane potential (MMP), and ultimately triggers mitochondrial-mediated apoptosis [24]. In order to explore whether shikonin induces cell apoptosis by inducing overproduction of ROS, we used DCFH-DA to label reactive oxygen species to evaluate the ROS level. The results showed that shikonin induces an increase in ROS (Figure 3A). In addition, we evaluated the downstream oxidative stress genes that regulate ROS production. The expression of nox1-1, glipr1b-2, and glipr1a-1 was significantly increased (Figure 2B), suggesting that shikonin can induce oxidative stress and increase ROS. Astaxanthin and fullerene (C60) are known to be able to reduce oxidative stress and inflammation [25,26,27]. Indeed, we found that astaxanthin can rescue hindered tail fin regeneration caused by shikonin (Figure 3B,D) and reduce the accumulation of ROS (Figure 3A).

Apoptosis is essential for the development and maintenance of life body, but due to certain factors, excessive apoptosis can cause autoimmune diseases or imbalanced body development [28]. As a tumour suppressor and a nuclear transcription factor, p53 can activate genes involved in cell apoptosis, cell cycle regulation and many other processes. When cells sense external apoptotic stress, cytoplasmic p53 transfers to mitochondria, binds to the anti-apoptotic Bcl-2 protein, releases pro-apoptotic Bax from the complex, and releases cytochrome C into the cytoplasm [29,30]. In order to detect cell apoptosis during the regeneration of juvenile tails, we collected shikonin-treated zebrafish larvae at 48 hpa, extracted the total RNA, and measured the mRNA expression levels of bax, bcl2, and p53 genes by real-time fluorescent quantitative PCR. The results showed that p53 and bax/bcl2 increased in a concentration-dependent manner (Figure 2C). We detected apoptotic cells in the wound at 6 hpa and 12 hpa, and the results indicated that shikonin promotes the increase of apoptotic cells in the wound (Figure 4A–C,E) but this can be effectively rescued with astaxanthin (Figure 4C). This suggests that shikonin induces the apoptosis of tail cells by overproducing ROS.

There is evidence that topical application of 50–100 μg shikonin has a certain inhibitory effect on intradermal histamine-induced capillary permeability and thermal injury-induced edema in rats [2]. the mechanism is that shikonin inhibit the permeability caused by intradermal histamine to suppress the edema of burns and promote the recovery of skin after burns [31]. In this experiment, we used shikonin to treat zebrafish larvae and our results show that shikonin inhibits caudal fin regeneration by upregulating oxidative stress and promoting apoptosis. Different results may be caused by the different mechanisms of Shikonin in different species.

In the inflammatory phase after caudal fin amputation, neutrophils are the main scavengers of small dead cell debris, and macrophages are essential to alleviate inflammation and support the normal regeneration of the regenerated caudal fin [10]. Our results showed that the number of neutrophils recruited after shikonin treatment and reduced in the early stage of wound repair at 6 hpa, but remained unchanged at 12 hpa, 24 hpa, and 48 hpa (Figure 5A,B). On the other hand, there was no obvious change in the macrophage level at all stages (Figure 5C). It can be therefore inferred that the early recruitment of neutrophils caused the apoptotic cells of the wound (Figure 4A–C), thereby hindering the fin regeneration in zebrafish larvae. In order to explore through which signaling pathway shikonin affects the regulation of fin regeneration, we adopted the AMPK inhibitor vidarabine in attempt to rescue the regeneration process. The results showed that vidarabine reduced the apoptotic cells induced by shikonin, and some of the reduced proliferating cells were also restored (Figure 6B–E). In addition, the phenotype of shikonin-hindered tail fin regeneration was rescued (Figure 6A).

Our research showed that shikonin inhibits fin regeneration, mainly because it induces oxidative stress and apoptosis, and inhibits cell proliferation. The AMPK signalling pathway plays a role in this process. The results suggest that shikonin should be used reasonably for wound repair and tissue regeneration.

## Data Availability

The study did not report any data.
